# High-Sensitive Ultraviolet Photodetectors Based on ZnO Nanorods/CdS Heterostructures

**DOI:** 10.1186/s11671-016-1818-6

**Published:** 2017-01-13

**Authors:** Kin-Tak Lam, Yu-Jen Hsiao, Liang-Wen Ji, Te-Hua Fang, Kai-Hua Hsiao, Tung-Te Chu

**Affiliations:** 1 Fujian University of Technology, Fuzhou, People’s Republic of China; 2 National Nano Device Laboratories, National Applied Research Laboratories, Tainan, 701 Taiwan; 3 Institute of Electro-Optical and Materials Science, National Formosa University, Yunlin, 632 Taiwan; 4 Department of Mechanical Engineering, National Kaohsiung University of Applied Sciences, Kaohsiung, 807 Taiwan; 5 Department of Mechanical Engineering and Automation Engineering, Kao Yuan University, Kaohsiung, 821 Taiwan

**Keywords:** CdS film, ZnO nanorods, Photodetectors

## Abstract

The ultraviolet (UV) photodetectors with ZnO nanorods (NRs)/CdS thin film heterostructures on glass substrates have been fabricated and characterized. It can be seen that the UV photoresponsivity of such a device became higher as the ZnO NR length was increased in the investigation. With an incident wavelength of 350 nm and 5 V applied bias, the responsivity of photodetectors based on ZnO NR/CdS heterostructures with the ZnO NR length at 500, 350, and 200 nm and traditional CdS film were at 12.86, 3.83, 0.91, and 0.75 A/W, respectively. The measurement results of the fabricated photodetectors based on ZnO nanorods (NRs)/CdS heterostructures have shown a significant high sensitivity in the range of UV light, which can be useful for the application of UV detection.

## Background

As we know, some pollution detection rely on ultraviolet (UV) spectroscopy, with detectors measuring the strength of absorption lines for such pollutants as ozone, nitrous oxides, and sulfur dioxide. High photoresponsivity and low noise levels are basically required for photodetectors in such applications [1, 2]. Because traditional silicon-based technology has some limitations in the UV photodetection. Recently, research and development in photodetectors have been mainly focused on nanostructures; a small absorption region with a high surface area to volume ratio not only contributes to a short transport time and thus higher response speed but also allows for better structural compatibility with scaling-down devices [3–6].

The bandgap energy of Si is 1.1 eV; costly high-pass optical filters and phosphors are needed to stop low-energy photons. Therefore, the most studied metal chalcogenide semiconductors, cadmium sulfide (CdS), exhibit a direct intermediate bandgap (∼2.5 eV) relatively low work function, large refraction index, and excellent thermal and chemical stability [7]. The fascinating material makes CdS one of the most important electronic and optoelectronic materials in nonlinear optical devices, photodetectors, waveguides, sensors, energy harvesting devices, or photoelectrochemical cells [8–11]. Photodetectors based on ZnO nanostructures have received extensive attentions due to their higher sensitivity in the UV range [12]. So far, a large variety of semiconductor nanostructures have been applied in photodetectors and some mechanisms involved have also been partly revealed [13–15]. Therefore, it will attract attention that the heterojunction photodetectors with ZnO nanostructures can be operated in the UV region. Therefore, the morphology and photoresponsivity properties of the ZnO nanowire on CdS thin film will be characterized because of their large surface area to volume ratio.

In this study, we synthesized the ZnO nanowire (NW)/CdS film heterostructure on glass substrates and then used such a structure to fabricate UV photodetectors. To our knowledge, CdS/ZnO NW heterostructures have not been thoroughly investigated yet for photodetectors, which was the motivation of this research. In this work, novel heterostructures made of CdS nanofilm and ZnO nanowires were prepared by a hydrothermal growth. Compared to pristine CdS nanobelts synthesized in the first stage of this work, CdS/ZnO NW heterostructures take obvious advantages over pure CdS nanofilm with respect to the ratio of photocurrent to dark current and responsivity.

## Methods

The CdS film was prepared according to the chemical bath deposition (CBD) procedure using cadmium chloride, thiourea [SC(NH_2_)_2_], and ammonium chloride, with purities of over 99.9%. Typically, aqueous solutions of 0.06 M CdCl_2_, 0.02 M SC(NH_2_)_2_, and 0.03 M NH_4_Cl were mixed in a glass beaker under magnetic stirring. The beaker was maintained at a reaction temperature of 70 °C using water bath. Various morphology films of CdS were deposited for 20, 30, and 40 min on the glass by CBD route. The various length of ZnO nanorods with 200, 350, and 500 nm were prepared by a two-step process. The 50-nm-thick ZnO seed layers were first deposited on CdS film by radio frequency (rf) magnetron sputtering. Then, we employed the photoresists in protecting the electrode patterns by lithography technique. Secondly, the sample with photoresist-protected interdigitated electrodes was subsequently immersed in the zinc nitrate: HMTA = 0.05 M:0.1 M aqueous solution for 1.0, 1.5, and 2.0 h at 90 ^o^C. The ZnO nanorod photodetectors were finished by removing the photoresists from the interdigitated electrode surface of devices. This synthesize was similar to our previous study [16]. The active area of the photodetector device was 150 × 160 μm^2^. The fingers of the Ag interdigitated (IDT) contact patterns were 150 μm long and 10 μm wide with 10-μm spacing.

**Fig. 1:**
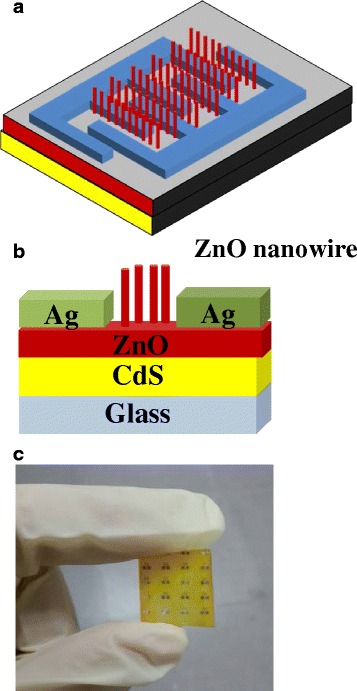
Schematic diagram of **a** top view and **b** cross section of ZnO nanorod on CdS film. **c** Optical image of ZnO nanorod on CdS film fabricated on glass

The schematic structure of the photodetector with ZnO NR/CdS film is shown in Fig. 1a. The various ZnO nanorod arrays on CdS film were examined by X-ray diffraction (XRD), field emission scanning electron microscopy (FE-SEM, JEOL JSM-6700F), high-resolution transmission electron microscopy (HRTEM, JEOL JEM-3010), and photoluminescence (PL, Labram HR). The photocurrent and dark current of photodetectors were then measured by a HP4156C semiconductor parameter analyzer at room temperature. Spectral responsivity measurements of the phototransistor were performed using a Jobin-Yvon SPEX system with a 300W Xe-arc-lamp light source (PerkinElmer PE300BUV) and a standard synchronous detection scheme at 300 Hz. The monochromator was covered the range of 300–600 nm. The area size of the illumination is larger than the detection area of the devices (~0.25 mm^2^). The obtained tendency is expected in this investigation; it can be seen that such a device with ZnO nanorod arrays has a good sensitivity in UVA (320–400 nm) range. The excitation light power mentioned in this work is referred to the actual light power shinning onto the device.

## Results and Discussion

**Fig. 2:**
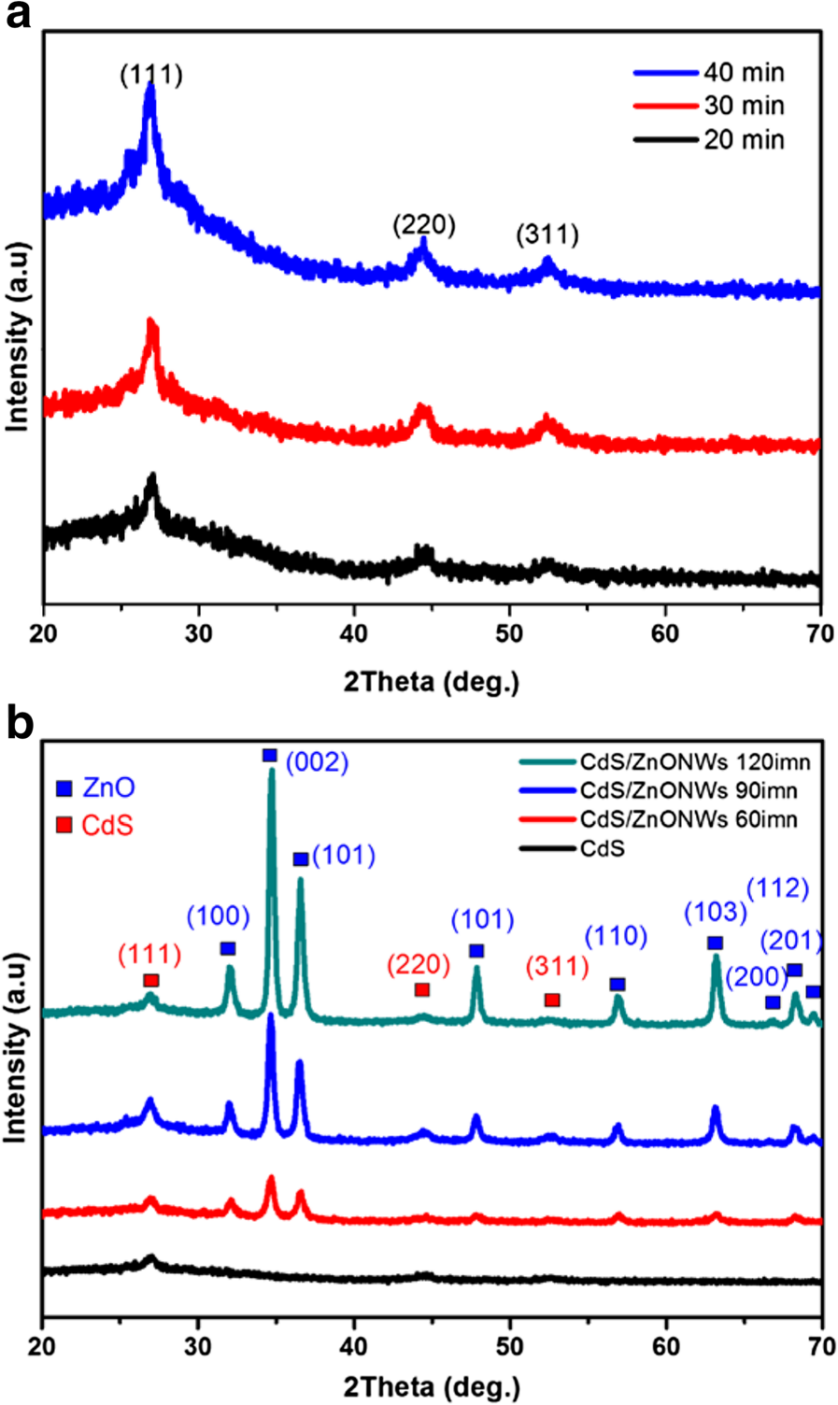
XRD pattern of **a** pure CdS film and **b** various length ZnO nanorods on CdS film

**Fig. 3:**
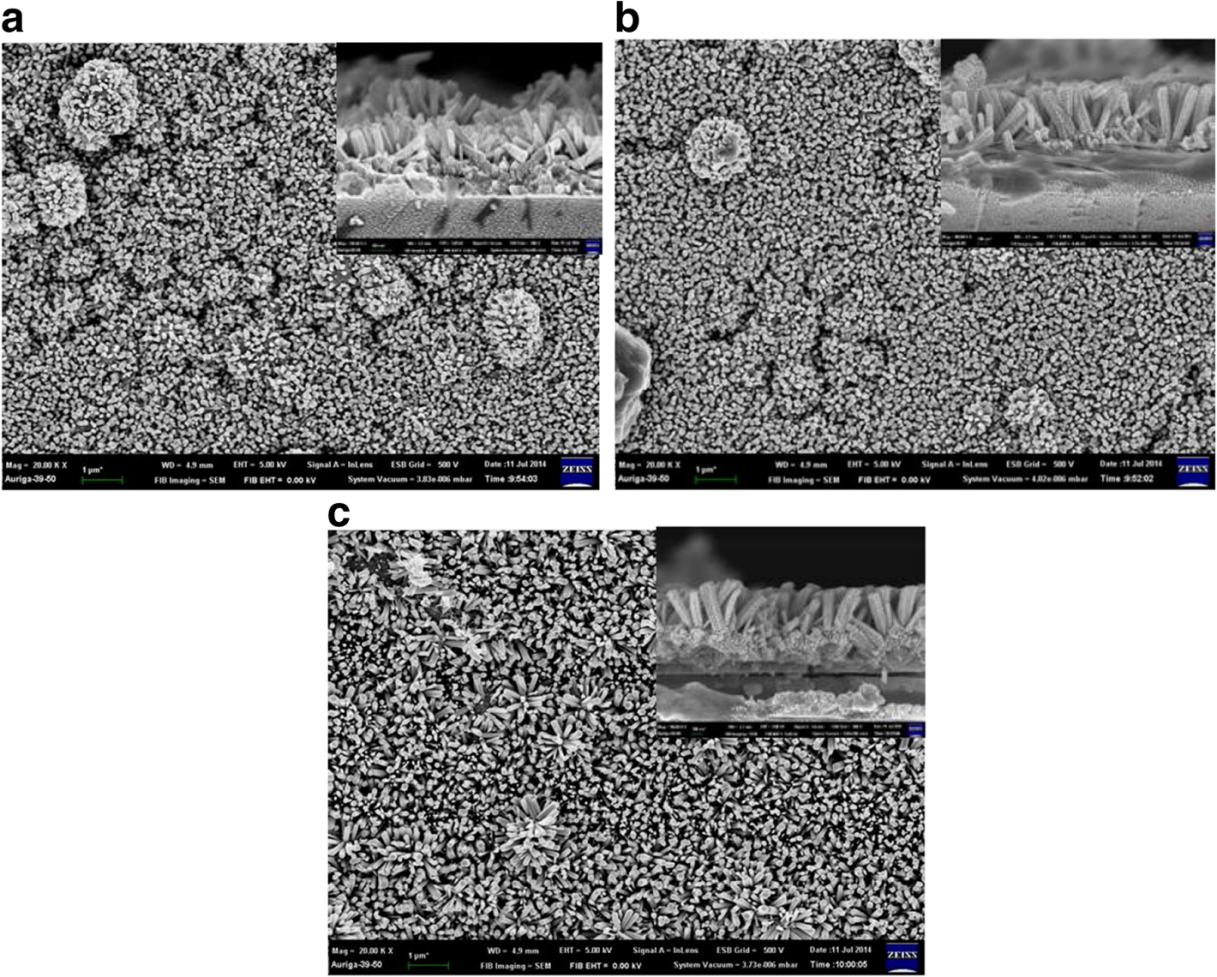
SEM top view of ZnO NWs with various length **a** 200, **b** 350, and **c** 500 nm on CdS film. The *inset* shows cross section of the ZnO NWs/CdS heterostructure

**Fig. 4:**
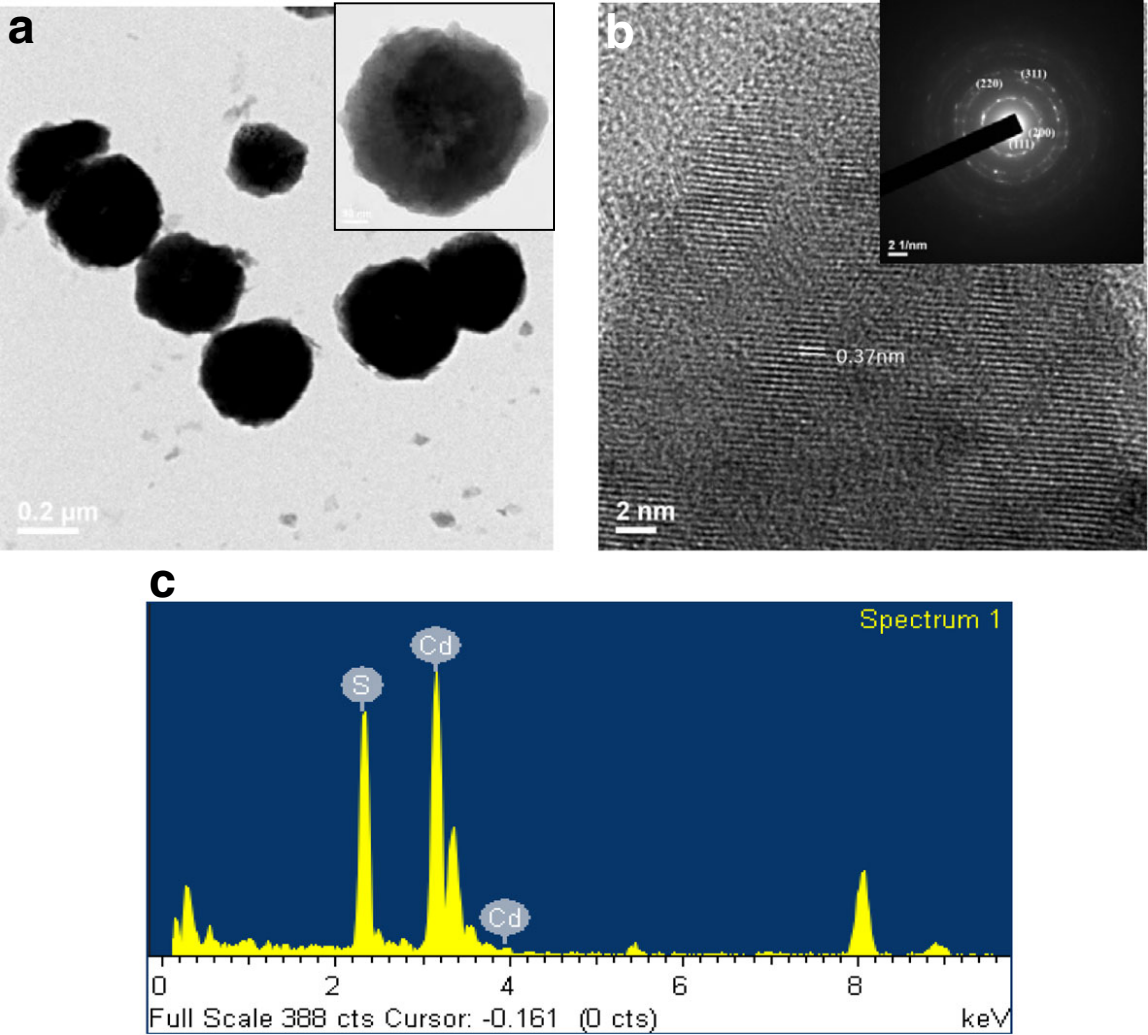
**a** TEM images of as-synthesized CdS nanocrystals. **b** High-resolution TEM image of the CdS nanocrystals and electron diffraction pattern. **c** EDS analysis of the CdS nanocrystals

The cross section of the photodetector device with ZnO nanorods/CdS heterostructure on glass substrate is shown in Fig. 1b. The as-fabricated photodetector was exhibited in Fig. 1c; it can be seen that the yellow of such a device is due to the native color of CdS. In Fig. 2a, XRD results of the CdS films were shown with deposited time 20, 30, and 40 min, respectively. It was found that the samples are with single crystal phase and all of the peaks were identified to be the cubic CdS phase (JCPDS #75-0581). The crystallinity of CdS film was increased with increasing deposited time. We used CdS film as the bottom layer with 40 min of deposited time. Figure 2b shows the XRD patterns of the zinc oxide nanorods on CdS/glass substrates with various growth time. It can be seen that the as-grown zinc oxide is with a polycrystalline structure and the intensity of the XRD peaks (100), (002), (101), (102), (110), (103), (200), (112), and (201) are increased while the growth time of ZnO NRs are increased. The peaks of CdS (111), (220), and (311) are not obviously increased. Figure 3a–c shows the SEM images of ZnO nanorods with 200, 350, and 500 nm were prepared by a two-step process. We found that the typical diameters of the ZnO nanorod was approximately to be 70 nm. It was found that ZnO nanowires (~200 nm) with irregular shape grown on the surface of CdS film as shown in Fig. 3a. As a result, the growth time of ZnO NRs is so short as to cover completely the surface of the cadmium sulfide grain, which can be proposed in this study. Figure 3c was shown that the ZnO nanorods (~500 nm) were distributed uniformly on entire substrate, and the hexagonal nanorods grew along c-axis direction perpendicular to the surface of the CdS film. The perpendicular behavior of ZnO NRs was similar to our previous investigation [17]. Figure 4a and the inset show a high-magnification TEM image of the crystal CdS and provide further insight into the nanostructured properties. The spherical morphology and uniform dispersion of CdS particle is clearly observed with the average diameter of 200 ~ 300 nm. The interplanar distances of the crystal fringes are estimated to be 0.37 nm, which is consistent with the (111) plane of CdS. As shown in the inset of Fig. 4b, the selected area electronic diffraction (SAED) pattern of the sample was also consistent with the characteristic of polycrystalline CdS. The EDS line profiles indicate that the film consists of cadmium and sulfur as shown in Fig. 4c. The atomic concentrations of Cd = 53.4% and S = 46.6% are calculated from the EDS spectrum.

**Fig. 5:**
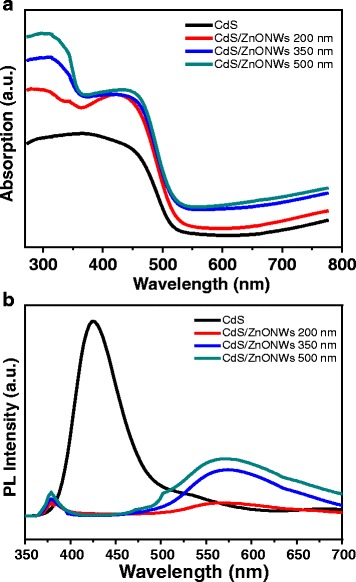
**a** Absorption and **b** photoluminescence (PL) spectra of the pure CdS film and various length ZnO NW/CdS heterostructures measured at room temperature

ZnO nanorod arrays on the CdS surface were observed by UV-visible spectrophotometer. We have estimated the bandgap energy from the absorption of the as-deposited CdS film in Fig. 5a. For a direct bandgap semiconductor, the absorbance in the vicinity of the onset due to the electronic transition is given by the following equation [18]:

1 $$ \alpha =\frac{C{\left(h\nu -{E}_g\right)}^{1/2}}{h\nu } $$

where *α* is the absorption coefficient, *C* is the constant, *hν* is the photon energy, and *E*_*g*_ is the bandgap energy. Extrapolation of the linear region gives a bandgap energy of 2.38 eV. The visible light absorption edge of the CdS film is located at 520 nm. Therefore, the direct bandgap energy is 2.38 eV in our investigation, and this is very close to the results reported recently by chemical bath deposition route [19]. As shown in Fig. 5a, it can be seen that the ultraviolet light absorption range between 280 and 370 nm can be contributed to ZnO nanorods. The absorptive intensity was increased as the thickness of the ZnO increases. It is possible that the crystallinity of the ZnO nanorod film became better; previous XRD results have confirmed this proposal.

The comparison on photoluminescence (PL) spectra between the CdS film and the ZnO NRs/CdS heterostructure are shown in Fig. 5b. A stronger PL peak centered at 428 nm was observed for the sample with a CdS film. On the other hand, it was found that a PL peak centered at 382 nm was revealed for a ZnO NW/CdS heterostructure film. As a result, it seem to be concerned with the ZnO composition. Furthermore, the broader PL peak from 475 to 700 nm (yellow band) could be probably attributed to the defects such as vacancies and stacking faults on the surface and interface of the ZnO NW/CdS heterostructure [20]. CdS heterostructure/CdS heterostructure is with lower emission intensity compared to the pristine CdS film, hence the recombination of the photogenerated carriers which was suppressed in such a heterostructure [21]. In addition, the higher PL emission intensity for ZnO NR (500 nm)/CdS heterostructure are in line with the higher structure crystallinity.

**Fig. 6:**
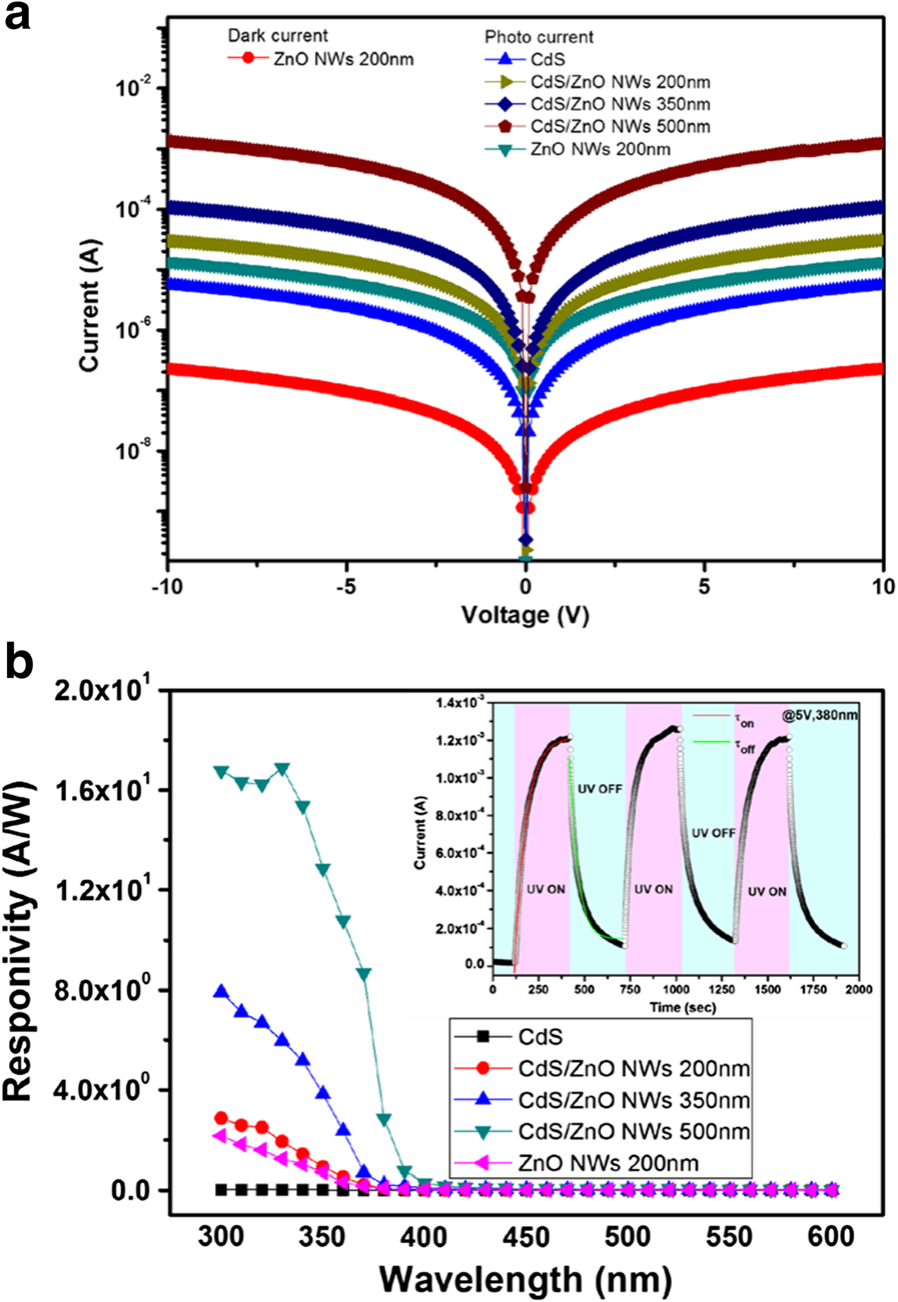
**a** Room temperature *I*–*V* characteristics of the various length ZnO NW/CdS heterostructure photodetectors under 350 nm UV illumination. **b** Photoresponsivity of the various length ZnO NW/CdS devices as a function of illumination wavelength. The *inset* shows time-resolved photocurrent of the length ZnO (500 nm) on CdS photodetector

Figure 6a shows the *I*–*V* characteristics of the photodetectors with different ZnO nanorods/CdS heterostructures layers at dark and illumination conditions. The measurements were under 5 V bias and 350 nm illumination. The data indicates that the light current of the photodetectors with increasing the length of ZnO nanorods were improved. The photocurrent to dark current contrast ratios of the ZnO nanorods (200nm)/CdS heterostructures based at 5 V were 146. The photodetection from ZnO NRs/nanowires of some important works have been done recently, which are summarize in Table 1 [22–25]. Figure 6b presents the photoresponsivity of the photodetectors with pure CdS fim, pure ZnO nanorod, and different length ZnO NR/CdS heterostructures in the range of UV-to-visible light. Photoresponsivity of the composited ZnO NR/CdS heterostructure device was shown as a function of illumination wavelength. It shows a decreasing response as the illumination wavelength is increased from 300 to 400 nm. We have demonstrated ZnO nanorods could be used as a material for the fabrication of ultraviolet photodetectors [16]. This result was attributed to the defects of ZnO nanorod surfaces which were the origin of the trap states, and the surrounding gas molecules will affect the changes on energy band and the dangling bonds [26, 27]. Furthermore, the high density of trap states caused an increase of carrier injection and transport and could produce a persistent photocurrent. The high responsivity of the photodetector based on ZnO NR (500 nm)/CdS heterostructure as compared with CdS can be attributed to the improved carrier collection efficiency. With an incident wavelength of 350 nm and 5 V applied bias, we found that photoresponsivity of devices with 500, 350, and 200 nm length ZnO NR and without ZnO NR on CdS film were 12.86, 3.83, 0.91, and 0.75 A/W, respectively. The inset of Fig. 6b shows the time-resolved response of the ZnO NW (500 nm)/CdS heterostructure, which the currents were measured by turning the UV light ON and OFF in atmosphere. By light illumination, photoexcited holes move to the surface and discharge the absorbed oxygen ions through electron-hole recombination. The unpaired electrons thus significantly enhance the conductivity. The oxygen adsorption and desorption process are usually slow, which lead to the slow response [28]. Thus, turn-ON speed should be slower than the turn-OFF speed for our ZnO NW (500 nm)/CdS photodetector. The turn-ON and turn-OFF transients can both be well fitted by the exponential curves. From the inset of Fig. 6b, it was found that the corresponding time constant for turn-ON transient was τON = 62.4 s, while that for turn-OFF transient was τOFF = 44.9 s. Such a result, we can see both of the turn-ON and turn-OFF transient time are large, because CdS with bandgap of 2.4 eV is a material of photoresistor, which its response speed is low in the UV range [21]. At this study, such an enhancement effect has also been studied in ZnS-coated ZnO nanowire arrays [29]. It can be expected that such a device will be used in the fabrication of high-sensitivity and flexible optoelectronic devices with possible fields of applications on low-light imaging sensors.

**Table 1:** Comparison of the photosensitivity reported in the literature and this work

Morphology	Device type	Light of detection (nm)	Bias (V)	Maximum photosensitivity	Ref
ZnO NWs/CdS film	Resistor	350	5	146	This study
ZnO nanorods	Resistor	325	2	19	[22]
ZnO nanowires	Resistor	254	5	17.7	[23]
ZnO NWs/paper	Resistor	360	10	80	[24]
AZO NWs:TiO_2_	Resistor	254	5	52	[25]

## Conclusions

In summary, we have investigated the ZnO NRs on the CdS film for the application of photodetection, which show higher response compared to the pure CdS film. As a result, it can be attributed to the high surface-to-volume ratios of ZnO nanostructure easily providing a carrier collection efficiency. With an incident wavelength of 350 nm and 5 V applied bias, we found that responsivity of ZnO nanowire length at 500, 350, and 200 nm and non-ZnO on CdS film were 12.86, 3.83, 0.91, and 0.75 A/W, respectively. The responsivity would become higher as ZnO nanorod length increased. It was found that the time constant for turn-ON transient was τON = 62.4 s and turn-OFF was τOFF = 44.9 s ZnO for NR (500 nm)/CdS heterostructure. The results exhibit significant improvement in the performance of photoinduced properties; this device will be useful in the field of UV detection.
